# Pragmatic failure, mind style and characterisation in fiction about autism

**DOI:** 10.1177/0963947014526312

**Published:** 2014-05

**Authors:** Elena Semino

**Affiliations:** Lancaster University, UK

**Keywords:** Autism, characterisation, fiction, figurative language, Grice, impoliteness, metaphor, mind style, pragmatic failure, Theory of Mind

## Abstract

This article presents an analysis of different types of pragmatic failure in the interactional behaviour of the ‘autistic’ protagonists of three recent novels. Three main types of pragmatic failure occur across all three novels: problems with informativeness and relevance in conversational contributions; problems with face management resulting in unintentional impolite behaviours; and problems with the interpretation of figurative language. These problems are salient and frequent enough to contribute to the projection of distinctive mind styles, and more generally to the characterisation of the protagonists as individuals with communication and socialisation difficulties that are likely to both reflect and reinforce general perceptions of autism-spectrum disorders. It is also argued that pragmatic failure contributes to the potential defamiliarisation of ‘normal’ communication, which is presented as being fraught with obscurity, ambiguity and insincerity.

## 1 Introduction

The last few decades have seen an increase in the presence of ‘autistic’ characters in different kinds of fiction, including television (e.g. the sit-com *The Big Bang Theory*, broadcast from 2007) and novels (e.g. Mark Haddon’s *The Curious Incident of the Dog in the Night-Time*, 2003) (see Bates, 2010; Greenwell, 2004). A variety of factors may be mentioned to explain this tendency. Autism-spectrum disorders (ASDs), including Asperger syndrome, were first described only in the 1940s (Asperger, 1944; Kanner, 1943).^1^ Since then, the steady rise in the number of diagnoses has progressively led to greater public awareness (Wing and Potter, 2002). More importantly for the purposes of this article, the difficulties in communication and socialisation associated with ASDs provide the opportunity to create fictional characters who take an estranged perspective on routine, everyday behaviours and activities, including particularly conversational interaction. In the first half of the 20th century, the Formalist school of criticism influentially described the defamiliarisation of familiar experiences as the main function of art generally and verbal art in particular (Shklovsky, 1965 [1917]). More recently, Cook (1994) restated the main claims of this approach to art and literature in terms of modern cognitive psychology: he argued that, in literary discourse, the use of distinctive linguistic patterns typically facilitates the ‘refreshment’ of readers’ existing schemata (see also Semino, 1995, 1997).

More specifically, the fictional representation of *minds* that work in distinctive and unusual ways has been noted both within narratology and literary stylistics. Margolin (2003: 287) points out a ‘preference of much literature for nonstandard forms of cognitive functioning, be they rare or marginal, deviant, or involving a failure, breakdown, or lack of standard patterns’. This, he argues, may in turn affect readers’ perceptions of the workings of their own minds. Within stylistics, Fowler’s (1977: 103) notion of ‘mind style’ as ‘any distinctive linguistic representation of an individual mental self’ has been applied to fictional narratives where a variety of linguistic patterns suggest ‘an unorthodox conception of the fictional world’ (Leech and Short, 1981: 188–189). The kinds of linguistic choices that have been associated with the projection of distinctive mind styles include patterns in lexis, syntax, transitivity, figurative language, speech representation and deixis (Bockting, 1995; Fowler, 1977, 1986; Leech and Short, 1981; Semino, 2002, 2011, 2014; Semino and Swindlehurst, 1996). Distinctive patterns in characters’ conversational behaviour in particular have been discussed in considerable depth in relation to characterisation in drama (e.g. Culpeper, 2001; Jeffries and McIntyre, 2010: 100ff.; Short, 1996: 195ff.). Some recent studies have also shown the potential relevance of fictional conversations to the projection of characters’ mind styles in narrative fiction, including particularly the mind styles of characters who may be described as ‘autistic’ (Fanlo Piniés, 2005; Semino, 2007, 2014).

In this article I draw from three recent English-language novels in order to show how the mind styles of autistic protagonists are conveyed, amongst other things, by difficulties in conversational interaction that can be described as different types of what Thomas (1983) calls ‘pragmatic failure’. In the three novels, pragmatic failure therefore contributes to the characterisation of the protagonists as individuals with the kinds of communication and socialisation difficulties that are associated with ASDs. I suggest that these fictional representations are likely to both reflect and reinforce general perceptions of ASDs.

Thomas (1983: 92) suggests that a speaker’s linguistic competence involves ‘grammatical competence’ and ‘pragmatic competence’, and describes the latter as ‘the ability to use language effectively in order to achieve a specific purpose and to understand language in context’. Pragmatic failure is therefore generally defined as ‘the inability to understand “what is meant by what is said”’ (Thomas, 1983: 91). Thomas is particularly concerned with the problems that a non-native speaker of a language may experience in cross-cultural communication (see also Jaworski, 2009), and makes a further distinction between two types of pragmatic failure:

a. *Pragmalinguistic failure*, which occurs when the pragmatic force mapped by S [speaker] onto a given utterance is systematically different from the force most frequently assigned to it by native speakers of the target language, or when speech act strategies are inappropriately transferred from L_1_ to L_2_.

b. *Sociopragmatic failure*, a term I have appropriated from Leech (1983: 10–11), which I use to refer to the social conditions placed on language in use.

(Thomas, 1983: 99)

In the novels under analysis, the characters with ASDs are neither non-native speakers of English nor outsiders to the culture they operate in. However, they are portrayed as experiencing various kinds of pragmatic failure, which can be interpreted as an indication that they lack, at least in part, the pragmatic competence that is normally associated with the ‘pragmatically intact speaker’ (Cummings, 2009: 14). These difficulties are among the clues that each novel provides in support of a more or less explicit attribution to the relevant character of some form of ASD.

More specifically, the different types of pragmatic failure experienced by the characters are likely to contribute to the projection of their mind styles, or, in Palmer’s (2004) terms, to inferences about the characters’ ‘fictional mental functioning’: these failures suggest that the three protagonists partly lack the ability, or motivation, to imagine the contents and workings of others’ minds. The attribution of mental states to others is crucial both to pragmatic theory and to ‘Theory of Mind’ problems, which are at the core of one of the main theories of ASD (e.g. Baron-Cohen, 1995; see Cummings, 2009: 119–121).^2^ In speech act theory, and, broadly speaking, in Gricean and neo-Gricean pragmatics, speakers’ intentions are central to the definition of notions such as illocutionary force and implicature: from the receiver’s perspective in particular, the recovery of illocutionary force, implicatures and many other types of inferences involves the attribution of intentions to a speaker or a writer. Intentions are of course a type of mental state, and the attribution of mental states to others is at the core of the concept of Theory of Mind – the ability to predict and explain other people’s behaviour in terms of their intentions, beliefs, desires, emotions, and so on. People with Theory of Mind deficits (or, as Baron-Cohen puts it, ‘mindblindness’) find it difficult to make sense of others’ actions and reactions by attributing mental states to them. This explains why, as Reboul et al. (2012: 317) put it, ‘[a]utism has been described as being to pragmatics what aphasia is to syntax, i.e., a natural testing ground for pragmatic hypotheses’ (but see Landa, 2009: 129 on the relative scarcity of empirical work on pragmatic functioning in ASDs).

Needless to say, the fictional representation of autism may not be, or aim to be, accurate from a clinical point of view. Nonetheless, while medical accuracy is not required for the success of a fictional narrative, stories involving characters with illnesses or disorders tend to be valued, among other things, for their degree of realism. This requires consistency, at least to some extent, with general perceptions of those conditions. On the other hand, successful fictional representations of particular phenomena, including cognitive disorders, may well contribute to shape readers’ knowledge and perceptions of real-life phenomena and people, and become part of public campaigns and debates (cf. Bates, 2010: 51). In addition, as mentioned earlier, the representation of characters with cognitive and/or communication difficulties can make explicit readers’ default assumptions about conversational interaction, and provide a fresh perspective on how readers perceive and evaluate ‘normal’ cognition and communication.

In the next section, I introduce the three novels under analysis. I then consider the three main types of pragmatic failure in turn: problems with informativeness and relevance, unintentional impoliteness, and difficulties with figurative language.

## 2 The three novels

The novels I discuss in this article were all published in the first decade of the 21st century. In chronological order of publication, they are: *Speed of Dark* by Elizabeth Moon (2002), *The Curious Incident of the Dog in the Night-Time* by Mark Haddon (2003), and *The Language of Others* by Clare Morrall (2008).

*Speed of Dark* is set in the USA during a hypothetical future time when autism is treatable in infancy or before birth, while gifted adults with autism are employed in specialized jobs thanks to their ability to detect complex patterns. Apart from a few stretches of third-person narration, the story is narrated in the first person by Lou, who describes himself as autistic. Lou works as a bioinformatics specialist in a pharmaceutical company where additional facilities are provided for autistic employees. As the story progresses, an experimental type of brain surgery becomes available to ‘cure’ autistic people like Lou, and he decides to undergo the operation. The end of the novel tells of Lou’s recovery and of his new life as a different person.

*The Language of Others* is set in Birmingham in present-day England. Jessica, the protagonist, grew up in a dilapidated stately home, trained as a musician and now works in a library, while still performing with her friend Mary from time to time. She narrates the parts of the novel where she is an adult, while her experiences as a child are narrated in the third person, mostly from her point of view. Jessica struggles socially as a child, and goes through life often feeling out of place, sometimes struggling to communicate successfully with others, and enjoying being alone more than in the company of other people. She goes through a difficult marriage and divorce with Andrew, a self-centred man with whom she has a son, Joel. Joel is extremely clever and passionate about computers, but also rather idiosyncratic and something of a loner. Eventually, Jessica is told by Joel’s fiancé that he has Asperger syndrome. This is a revelation for Jessica, which makes her wonder about herself too.

*The Curious Incident of the Dog in the Night-Time* (henceforth *The Curious Incident*) is set in Swindon in present-day England. The protagonist and first-person narrator is 15-year-old Christopher, who goes to a special-needs school and describes himself as having ‘behavioural problems’. Christopher is a talented mathematician and explains that he has reluctantly agreed to write a novel at the invitation of his favourite teacher Siobhan. However, as he does not like ‘proper novels’, he has decided to write an autobiographical murder mystery novel, in which he aims to chronicle his attempt to discover who killed his next-door neighbour’s dog, Wellington. As the story progresses, Christopher not only discovers the identity of Wellington’s murderer but much else besides, and goes through several traumatic experiences (Semino, 2011, 2014).

The three novels are sufficiently similar to be compared with one another, but also sufficiently different to provide a range of fictional representations of ASD. All three protagonists are at the high-functioning end of the autistic spectrum, and all three have a special ability that can be seen as the flip-side of their impairments in communication and socialisation: both Lou and Christopher have mathematical skills that are reminiscent of savants, and Jessica, while not a prodigy, is a talented musician. Haddon’s novel has in fact been criticised for reinforcing the mistaken perception that people with autism tend to have savant qualities (Draaisma, 2009), and the same criticism could potentially be levelled against *Speed of Dark* in particular.

As for differences among the three novels, *Speed of Dark* is set in the USA in the near future, while the other two novels are set in present-day England. *Speed of Dark* has an adult protagonist, *The Curious Incident* a teenage protagonist and *The Language of Others* portrays the protagonist as both a child and an adult. In *Speed of Dark*, autism is a central theme and the protagonist’s relationship with his own autism is at the centre of the plot. In *The Language of Others*, the protagonist’s mother wonders about her being autistic early on, but she is never diagnosed, and only hears about her son’s Asperger syndrome towards the end of the novel. In *The Curious Incident* the protagonist is clearly diagnosed as having special needs in the fictional world but the words ‘autism’ and ‘Asperger’ never occur. Nonetheless, Christopher is normally described in these terms by readers, reviewers, critics, Haddon himself, and the book’s back cover. *The Curious Incident* is narrated entirely in the first person by Christopher; *Speed of Dark* is mostly narrated in the first person by Lou; *The Language of Others* alternates between first- and third-person narration.

## 3 Informativeness and relevance

All three novels suggest that the protagonist finds it difficult, at least some of the time, to infer other characters’ unstated meanings and intentions, as well as to provide contributions that are cooperative or optimally relevant from the perspective of their interlocutors, and from the likely perspective of readers. These difficulties suggest that the three characters are often unable to ‘read’ others’ minds successfully, and hence contribute to the projection of mind styles that can be associated with an autistic-spectrum disorder.

In the following extracts, from *Speed of Dark*, Lou narrates an interaction with a new manager in his company, Mr Crenshaw, who is opposed to the ways in which the company accommodates the needs of autistic employees:

(1)  Mr Crenshaw comes up again at closing time, when I am still working. He opens my    door without knocking. I don’t know how long he was there before I noticed him, but I    am sure he did not knock. I jump when he says ‘Lou!’ and turn around.

‘What are you doing?’ he asks.

‘Working,’ I say. ‘What did he think? What else could I be doing in my office, at my workstation? (*Speed of Dark*, p. 25)

(2) […] ‘You started late today,’ he says.

‘I’m staying late tonight,’ I say. ‘I was one hour and forty-seven minutes late. I worked through lunch; that is thirty minutes. I will stay one hour and seventeen minutes late.’

‘You’re honest,’ he says, clearly surprised.

‘Yes,’ I say. I do not turn to look at him. I do not want to see his face. After seven seconds, he turns to leave. (*Speed of Dark*, p. 27)

In (1) Lou appears to be puzzled by Mr Crenshaw’s question (‘What are you doing?’), and provides an answer that could be described as not sufficiently informative. In (2) Lou’s references to time may be described as unnecessarily detailed, both when he talks to Mr Crenshaw and when, as a narrator, he specifies the number of seconds that intervened between his own last turn and Mr Crenshaw leaving.

Something similar can be observed in Extract (3), from *The Curious Incident*. Christopher is being questioned by the police, who have been called by Christopher’s neighbour, Mrs Shears, after she discovered Christopher in her garden in the middle of the night holding the body of her dog with a garden fork sticking out of its stomach.

(3)  […] The policewoman put her arms round Mrs Shears and led her back towards the house.

I lifted my head off the grass.

The policeman squatted down beside me and said, ‘Would you like to tell me what’s going on here, young man?’

I sat up and said, ‘The dog is dead.’

‘I’d got that far,’ he said.

I said, ‘I think someone killed the dog.’

‘How old are you?’ he asked.

I replied, ‘I am 15 years and 3 months and 2 days.’ (*The Curious Incident*, p. 7)

The first two utterances Christopher produces in the interaction with the policeman may be described as insufficiently informative. In contrast, in the final utterance of the extract, Christopher provides his age with unnecessary precision.

The peculiarities of Lou’s and Christopher’s communicative behaviour can be accounted for in terms of Grice’s Cooperative Principle and its developments within neo-Gricean pragmatics. In Grice’s (1989) terms, both characters seem to have difficulties with the maxim of quantity:

The category of Quantity relates to the quantity of information to be provided, and under it fall the following maxims:

1. Make your contribution as informative as is required (for the current purposes of the exchange).
2. Do not make your contribution more informative than is required. (Grice, 1989: 26)

The first sub-maxim is broken by Lou’s response in (1) and Christopher’s utterances about the dog in (3): Mr Crenshaw can see or reasonably expect Lou to be working while sitting at his computer terminal in his office; and the police have been called by Mrs Shears precisely because it is clear that her dog has been killed. The second sub-maxim is broken by both Lou’s and Christopher’s over-informative references to time, whether in dialogue or in narration: none of these details turns out to be significant in making sense of the current scene or of later plot developments. Arguably the latter type of utterance also breaches one of Grice’s Manner maxims (‘Be brief’) and the maxim of Relation (‘Be relevant’).

In Levinson’s (2000) terms, the over-informative contributions breach the speaker’s maxim under the I-Principle: ‘Say as little as necessary’ (Levinson, 2000: 114). The uninformative contributions, in contrast, breach the speaker’s maxim under the Q-Principle: ‘Do not provide a statement that is informationally weaker than your knowledge of the world allows, unless providing an informationally stronger statement would contravene the I-Principle’ (Levinson, 2000: 76. See also Horn, 2004).^3^

From both Grice’s and Levinson’s perspectives, breaches of maxims and principles tend to be explained in terms of speakers’ intentions, such as the intention to deceive, obfuscate, convey implicatures, and so on. In my examples, however, Lou’s and Christopher’s breaches of the Quantity sub-maxims, and of Levinson Principles, do not appear to reflect the intention to deceive or convey implicatures. Rather, they are likely to be interpreted as a result of the characters’ inability to assess what their addressee needs to know, whether the relevant addressee is another character in the fictional world or the imaginary recipients of their narratives. In character–character interaction, the two protagonists’ breaches of the first Quantity sub-maxim occur in response to questions that appear to be quite general: ‘What are you doing?’ and ‘Would you like to tell me what’s going on here, young man?’ Neither character seems to assess the questioner’s intentions and existing knowledge successfully enough to provide an optimally informative answer.

Thomas’s (1995) definition of ‘infringement’ in relation to Grice’s maxims is particularly useful in order to capture the way in which Lou and Christopher break the two Quantity sub-maxims (see also Culpeper, 2001: 143):

A speaker who, with no intention of generating an implicature and with no intention of deceiving, fails to observe a maxim is said to ‘infringe’ the maxim. In other words, the non-observance stems from imperfect linguistic performance rather than from any desire on the part of the speakers to generate a conversational implicature. This type of non-observance could occur because the speaker has an imperfect command of the language (a young child or a foreign learner), because the speaker’s performance is impaired in some way (nervousness, drunkenness, excitement), because of some cognitive impairment, or simply because the speaker is constitutionally incapable of speaking clearly, to the point, etc. (Thomas, 1995: 74)

Competent readers of the two novels are likely to infer, in each case, that the character’s/narrator’s communicative behaviour reflects a cognitive impairment that they may identify as high-functioning autism or Asperger syndrome. At the level of character–character communication, however, the characters’ responses are sometimes perceived to be puzzling or deliberately uncooperative, resulting in misunderstanding and/or conflict.

In terms of Relevance Theory (Sperber and Wilson, 1986, 1995), the two protagonists’ communicative behaviour in the extracts given earlier seems to fall short of the Communicative Principle of Relevance, according to which ‘[e]very ostensive stimulus conveys a presumption of its own optimal relevance’ (Wilson and Sperber, 2004: 612). Strictly speaking, the communicative contributions under analysis do not provide sufficient contextual effects to justify the effort involved in processing them, either because they are overly detailed or because they are not detailed enough. From the reader’s point of view, however, the processing effort is in fact justified by inferences about the protagonists’ cognitive abilities. Wilson and Sperber’s detailed definition of the ‘presumption of optimal relevance’ accounts for this:

**Presumption of optimal relevance**

- The ostensive stimulus is relevant enough to be worth the audience’s processing effort.
- It is the most relevant one compatible with the communicator’s abilities and preferences. (Wilson and Sperber, 2004: 612)

Arguably, in the extracts given earlier, the ostensive stimuli produced by Lou and Christopher are indeed the most relevant ones compatible with their abilities. Inferences about their abilities are central to the interpretation of each novel, as are inferences about the abilities that tend to be taken for granted when communication flows smoothly: readers may have the opportunity to reflect on the potential vagueness and ambiguity of frequent and mundane questions such as ‘What are you doing?’ or ‘What’s going on?’

In *The Language of Others*, Jessica does not display this kind of difficulty quite to the same extent as Lou and Christopher. However, she is occasionally hampered by uncertainty about what someone else meant. In the following extract, Jessica and her father are showing her boyfriend Andrew around their large house, Audlands Hall.

(4)  ‘So which bit do you live in?’

I couldn’t decide what he meant. Was he talking about me personally? My bedroom? Or which bit did we all live in? What did he mean by ‘live’? The kitchen? Or the drawing room? I didn’t know how to answer.

‘We live in all of it,’ said my father.

‘Really?’ said Andrew. ‘Wow.’ (*The Language of Others*, p. 24)

Jessica’s frantic thoughts after Andrew’s question show that she cannot easily infer what exactly he wishes to know. She therefore delays providing an answer long enough for her father to answer on her behalf.

In Thomas’s (1983) terms, the extracts I have discussed in this section involve pragmalinguistic failure in the sense that, on the one hand, the three characters seem unable to understand fully what is meant by what others say, while, on the other hand, both Lou and Christopher do not appear to be fully aware of what meanings their interlocutors may infer from what they themselves say. These failures contribute to the projection of mind styles that may be associated with an ASD: readers are put in a position to infer that all three characters struggle to guess other characters’ existing knowledge and mental states, and are therefore sometimes unable to interact effectively. Indeed, a number of studies have suggested that real-life people with high-functioning autism and Asperger syndrome have more difficulties than neurotypical controls to make some kinds of inferences, especially by exploiting the available context (e.g. Loukusa et al., 2007; Pijnacker et al., 2009).

The Gricean maxim that, in contrast, tends to be fulfilled in the strictest sense in the foregoing extracts, and more generally in the three novels, is the Maxim of Quality (‘Try to make your contribution one that is true’; Grice, 1989: 27). Following on from Grice’s (1989: 371) own observations, in neo-Gricean pragmatics, truthfulness is indeed treated as separate from the principles that concern informativeness and relevance. Christopher explicitly says that he does not ‘tell lies’, not because he is a ‘good person’ but because he ‘can’t tell lies’ (*The Curious Incident*, p. 24). In Extract (2), Lou’s honesty is explicitly mentioned in the conversation. Jessica is often puzzled by others’ lies, and she mentions her friend Mary’s advice to her that ‘Honesty isn’t always the best policy’ (*The Language of Others*, p. 156). This is broadly consistent with the attribution of Theory of Mind problems to the three characters, as lying involves the deliberate manipulation of others’ beliefs, or, more precisely, the attempt to generate in others a belief that one knows is false. There is indeed some evidence that real-life people with ASDs engage less frequently and successfully than normal controls (e.g. Talwar et al., 2012). In the next section I consider the consequences of the characters’ approach to truth-telling for their ability to manage the face needs of others.

## 4 Impoliteness

The extracts I discuss in this section show how, in all three novels, the protagonists’ rather relentless approach to sincerity affects their ability to manage other characters’ ‘faces’ in interaction, and can result in unintentional face damage (Brown and Levinson, 1987; Goffman, 1967). This is consistent with the attribution to all three characters of the kind of Theory of Mind problems that are associated with ASDs.

In the following extract from *Speed of Dark*, Lou is talking to Mr Stacy, the police officer who is dealing with repeated incidents of damage to Lou’s property. Lou has just performed some mental arithmetic at a speed that strikes the police officer as exceptional:

(5) He is staring at me now with his mouth a little open. ‘You – calculated that? In your head?’

‘It is not hard,’ I say. ‘It is simply a permutation problem, and the formula for permutations is taught in high school.’

[…]

‘Good God,’ he says. ‘You’re serious.’ He shakes his head abruptly. ‘Sorry. I hadn’t – I didn’t know you were a math genius.’

‘I am not a math genius,’ I say. I start to say again that these calculations are simple, within the ability of school-children, but that might be inappropriate. If he cannot do them, it could make him feel bad. (*Speed of Dark*, pp. 239–240)

Lou modestly dismisses Mr Stacy’s surprise at his mathematical abilities by saying what he believes to be true, namely that the type of calculations he has just performed are taught in high school. When he is about to state this for the second time, he realises that this could make Mr Stacy ‘feel bad’, or, in Brown and Levinson’s (1987) terms, damage his positive face, as he would implicitly be described as unable to do what schoolchildren are expected to do.

In Extract (6) from *The Curious Incident*, Christopher is reporting a conversation with his teacher Siobhan:

(6) I also said that I cared about dogs because they were faithful and honest, and some dogs were cleverer and more interesting than some people. Steve, for example, who comes to school on Thursdays, needs help to eat his food and could not even fetch a stick. Siobhan asked me not to say this to Steve’s mother. (*The Curious Incident*, p. 6)

Christopher’s unfavourable comparison between one of his school friends and a dog would be extremely face-threatening if uttered in the presence of someone who is very close to Steve. Siobhan’s reported invitation to Christopher not to make this comment to Steve’s mother suggests that she fears that Christopher could potentially do just that, without considering the consequences that this may have for her feelings.

In Extract (7), the first of two extracts from *The Language of Others*, Jessica is talking to her boyfriend’s parents during their first meeting in a Chinese restaurant. The topic is Audlands Hall, the dilapidated stately home in which Jessica grew up.

(7)  ‘We’ve admired it [Audlands Hall] from the road,’ said Donald.

‘You can’t see it properly,’ I said, ‘unless you go up the drive.’

Miranda leaned forward as if she was going to say something important. ‘We went up the drive – just a little way – to have a peep. It’s beautiful, isn’t it?’

They had trespassed on our property. I thought of them driving in, going halfway up the drive as if they lived there. How would they like it if my family drove into their garden and peered through the windows? ‘It’s a private drive,’ I said with indignation.

She sat back, surprise making her eyelids open wider. (*The Language of Others*, pp. 61–62)

Jessica feels insecure and protective about Audlands Hall, and is perturbed by the idea of Miranda and Donald scrutinising it. Her response to Miranda ‘It’s a private drive’ is consistent with the Maxim of Quality but arguably flouts the maxims of Quantity and Relation by saying something that is either already known to the addressees or not relevant to their interest in the house. This flout conveys a face-threatening implicature, namely that Miranda and Donald should not have driven up the drive to take a better look at the house. In the social context of the dinner, it would have been more appropriate for Jessica not to convey her feelings at all. However, Jessica only seems to realise that she had ‘said something wrong’ afterwards, from Miranda’s and Andrew’s reactions.

In Extract (8), Jessica is going for a drink with her musician friend Mary and their agent Stuart:

(8)  We drive into the city centre and head for the bar at the Repertory Theatre. We find a table and sink down with relief.

‘When are you going to get a haircut?’ I say to Stuart. ‘It’s looking more porcupine than hedgehog.’

‘Jess …’ says Mary in a low voice, shaking her head very gently.

But Stuart doesn’t mind. He laughs. ‘Wait until I can’t get through doorways. Then you can worry.’ (*The Language of Others*, pp. 157–158)

Here Jessica volunteers a critical comment about Stuart’s hair which is humorous but also face-threatening, partly because it is uncalled for. Mary’s reaction suggests that she feels that the remark is inappropriate but Stuart seems to accept Jessica’s well-intentioned criticism and responds equally humorously.

What can be observed for all three protagonists is the potential to be unintentionally impolite, partly as a result of privileging sincerity over the face needs of others. Culpeper (2011) defines impolite behaviours as follows:

Situated behaviours are viewed negatively – considered ‘impolite’ – when they conflict with how one expects them to be, how one wants them to be and/or how one thinks they ought to be. Such behaviours always have or are presumed to have emotional consequences for at least one participant, that is, they cause or are presumed to cause offence. Various factors can exacerbate how offensive an impolite behaviour is taken to be, including for example whether one understands a behaviour to be strongly intentional or not. (Culpeper, 2011: 23)

In Extract (6) Christopher is implicitly attributed the tendency to say things that can have negative emotional consequences for others. In Extract (5) Lou becomes aware of the potential offensiveness of his utterances as he is producing them, and restrains himself before going too far. In Extracts (7) and (8) Jessica does not seem to be aware of the potential offensiveness of her candid remarks until she sees the reactions of others.

In all three novels, the protagonists potentially cause offence not out of malice, but because of a partial lack of awareness of the potential consequences of what they say for others’ feelings, and, in turn, for their own social image. This is broadly consistent with a Theory of Mind problem, as it suggests a difficulty in imagining others’ internal states, and especially emotional reactions. In fact, these cases are particularly consistent with the view that people with ASDs are not cognitively *deficient* with respect to Theory of Mind, but rather lack the *motivation* to attend to others’ minds, including others’ emotions (Hobson, 2012). In Thomas’s (1983) terms, this results in the kind of pragmatic failure that she calls ‘sociopragmatic’, as the characters fail to observe the social expectations prevalent in their culture about what one can appropriately say to others without jeopardising good social relations (see also Marmaridou, 2011). While discussing the pragmatic difficulties of people with high-functioning autism and Asperger syndrome, Landa (2009: 131) indeed observes that ‘opinions may be expressed forthrightly rather than in more subtle, socially acceptable indirect ways, leading to impressions of impoliteness, rudeness, or insensitivity’.

While these impolite behaviours can lead to inferences about the workings of the characters’ minds, they do not seem likely to result in negative characterisation. This is for three main reasons. First, from the vantage point of readers, the three protagonists do not, as I have mentioned, appear to *intend* to cause offence to other characters. As such, the utterances I have discussed in this section would not in fact be described as ‘impolite’ within approaches to impoliteness where intentionality is a necessary property of impolite behaviours (e.g. Bousfield, 2008: 72). According to Culpeper’s (2011) definition just given, impolite behaviours do not have to involve the intention to offend, but the attribution of intentionality is one of the factors that may ‘exacerbate how offensive an impolite behaviour is taken to be’ (Culpeper, 2011: 23).

The second reason why the three characters’ impolite behaviours are unlikely to lead to negative characterisation is closely linked to the first. Within each fictional world, nobody gets hurt very much by the protagonists’ impoliteness, or, if someone does, readers are unlikely to sympathise with them. Lou restrains himself just before inadvertently portraying his interlocutor as a very poor mathematician. Christopher, who could seriously hurt Steve’s mother’s feelings, is not presented as actually doing so in the fictional world. In (8) Stuart does not appear to be offended by Jessica’s remark. In contrast, in (7) Miranda appears to be taken aback by Jessica’s utterance, but in the previous turns Miranda’s interest in Audlands Hall seems to have been motivated by some degree of jealousy and snobbery linked to social class. Readers are therefore unlikely to have much sympathy for her. In fact, in all cases the real or potential faux pas that the characters are involved in are potential sources of humour. Finally, extracts such as the ones I have quoted contribute to the presentation of routine interactions as social minefields: readers are likely to become more aware of how difficult it is to judge what can and cannot be said, and particularly of a potential conflict between sincerity in interaction and the maintenance of social harmony.

## 5 Literal and figurative meanings

In all three novels, there are occasions when the protagonist struggles to make sense of non-literal language, and especially metaphors. This also contributes to the projection of mind styles that can be associated with an ASD. In both *Speed of Dark* and *The Curious Incident*, the narrator explicitly reflects on how some metaphorical expressions do not make sense to him. In (9) and (10) following, Lou comments respectively on the current conversation with his friend Marjorie and a remembered conversation with a neighbour he often meets in the shared laundry room of his apartment block:

(9)   ‘Tom and Lucia both sounded angry with Don,’ I say.

[…]

‘Don can be a real heel,’ she says.

Don is not a heel; he is a person. Normal people say things like this, changing the meaning of words without warning, and they understand it. […] If someone is a bad person, why not just say it? (*Speed of Dark*, p. 37)

(10)   ‘I need to go make some phone calls; are you going to be here? To watch the dryer?’

‘I will be downstairs,’ I say. ‘Not in this room; it is too noisy.’ I have said this before when she has asked me to keep an eye on her clothes. I always think of taking out an eye and putting it on the clothes, but I do not tell her this is what I think. I know what that expression means socially, but it is a silly meaning. (*Speed of Dark*, pp. 153–154)

In *The Curious Incident*, Christopher mentions that one of the two main reasons why he finds people ‘confusing’ is that

(11)   […] people often talk using metaphors. These are examples of metaphors

**I laughed my socks off.**

**He was the apple of her eye.**

**They had a skeleton in the cupboard.**

**We had a real pig of a day.**

**The dog was stone dead.**

[…]

I think it [metaphor] should be called a lie because a pig is not like a day and people do not have skeletons in their cupboards. And when I try and make a picture of the phrase in my head it just confuses me […]. (*The Curious Incident*, pp. 19–20; bold in original)

A little later in the novel, Christopher reports being confused by similar cases of metaphorical idioms:

(12) And she said lots of things I didn’t understand, e.g. ‘I’m going to hit the hay,’ and, ‘It’s brass monkeys out there,’ and ‘Let’s rustle up some tucker.’ And I didn’t like it when she said things like that because I didn’t know what she meant. (*The Curious Incident*, p. 55)

In *The Language of Others*, Jessica is confused by the humorous metaphors that her boyfriend, Andrew, uses to describe his own mother:

(13) ‘You won’t like my mother,’ said Andrew on the day I was going to meet his parents for the first time. ‘She’s the stepmother from Cinderella, the Queen in Snow White, a female Pol Pot.’

I couldn’t work out if he was joking. ‘Did she beat you?’

‘It was a beating of the mind, the spirit.’

[…]

‘My mother is Lucrezia Borgia,’ said Andrew. […] ‘Mrs Ceauşescu, Imelda Marcus without the shoes.’

‘Doesn’t your mother wear shoes?’

He rolled his eyes. (*The Language of Others*, pp. 55–56)

There is evidence that real-life people with ASDs have problems with some types of figurative language, including metaphor (e.g. Happé, 1995). Broadly speaking, this is explained as a consequence of more general difficulties with inferring speakers’ meanings from what is said. According to some recent research, the difficulties experienced by people with some forms of ASD in fact parallel those experienced by members of control groups: for example, all groups have more problems understanding novel metaphors than conventional metaphors (Giora et al., 2012). Nonetheless, these studies confirm the fact that people with ASDs have greater difficulties with figurative language than their neurotypical counterparts.

The foregoing examples are in fact among the few very salient occasions in each novel where the protagonist has a problem with metaphorical language. As readers cannot necessarily be expected to have the requisite metalinguistic awareness, in *Speed of Dark* and *The Curious Incident* the narrators explicitly spell out what kinds of expressions they find difficult and why; in *The Curious Incident*, Christopher explicitly talks about metaphor and contrasts it with simile, which he prefers because it ‘is not a lie’ (*The Curious Incident*, p. 22). The actual expressions that each of the three protagonists struggles with are also very obvious instances of figurative language, namely: conventional and relatively non-transparent metaphorical idioms in Examples (10), (11) and (12); and ‘equative’ metaphors in (9) and (13), in other words, metaphorical statements that take the form ‘X is Y’. The latter are not the most frequent type of metaphor in general language use, but they tend to be the most obvious ones: they involve blatantly ‘untrue’ statements and metaphorically used nouns, which, as Goatly (1997: 83) puts it, are ‘more recognisable as metaphors’ than metaphorical uses of other word classes. In Extract (13), Andrew’s descriptions of his mother are also highly creative and humorous. This arguably makes Jessica’s confusion understandable and plausible, while also drawing attention to her limitations as a competent adult speaker of the language.

More specifically, in these extracts Christopher and Jessica appear unable to disregard the literal meanings of metaphorical utterances, and experience confusion as a result of that. In Extracts (9) and (10), Lou actually understands what is intended by the relevant expressions but, like Christopher, he reports being distracted and frustrated when he attempts to imagine the literal scenarios and make sense of them. Elsewhere in *Speed of Dark*, however, Lou actually interprets some metaphorical expressions literally. In Thomas’s (1983) terms, these difficulties fall at the pragmalinguistic end of pragmatic failure (see also Marmaridou, 2011). Both Lou and Christopher also make negative judgements on metaphorical language, on the grounds that it fails on their strict approach to clarity and truthfulness.

In all cases, the characters’ difficulties draw attention to the literal nonsensicality of some figurative expressions, once again adding interest and humour to the characters’ interactions and reactions. Not surprisingly, however, in all three novels, the fictional representation of problems with metaphors is only as consistent as is required by the production of readable and effective narratives: any story told by someone who struggles with all or most uses of metaphors would be quite a demanding and frustrating read. All three narrators/characters do in fact produce and understand many instances of figurative language. The presence of extracts such as those I have given is arguably sufficient to establish that the three protagonists have some kind of cognitive impairment that makes it unusually difficult for them to interpret figurative language.

## 6 Concluding remarks

In this article I have discussed three novels in which the protagonist is attributed, more or less explicitly, an autism-spectrum disorder. My analyses have shown how, in these novels, the distinctive mind styles of the three characters are conveyed, in part, via three main types of pragmatic failure in reported conversations: problems with informativeness and relevance, unintentional impoliteness, and difficulties in the interpretation of figurative language. I have also suggested that the problems experienced by the characters are consistent with deficiencies in their Theory of Mind abilities or inclinations.

For reasons of space, I have not discussed other linguistic choices and patterns that also contribute to the projection of ‘autistic’ mind styles in each of the three novels, nor have I accounted for the differences in the mind styles that readers are likely to attribute to each of the three protagonists (but see Semino 2014 for a more comprehensive analysis of *The Curious Incident*). By focusing on pragmatic failure, however, I have shown how peculiarities in conversational behaviour can be exploited in the creation of distinctive mind styles. I have also suggested that the three kinds of pragmatic failure do not occur consistently and systematically in each novel, but are salient and frequent enough to put readers in a position to infer that the character has some of the cognitive and communicative difficulties that are associated with ASDs. This observation is not intended to undermine Fowler’s (1977: 76) claim that mind styles result from the cumulative effect of ‘consistent’ linguistic patterns. Rather, I am suggesting that, when a particular (linguistic) behaviour is foregrounded through ‘deviation’ from default or conventional expectations, a few instances of that behaviour may be sufficient to attribute a (mental) trait to a character, even though that character does not exhibit that behaviour consistently (see also Culpeper, 2001: 128ff).

Although I have, in the course of the discussion, referred to some relevant studies on real-life people with ASDs, I have not been directly concerned with the medical accuracy of the three fictional representations of autism. While all three protagonists exhibit some of the characteristics associated with real-life people with ASDs, I would not wish to claim that any of them are accurate representations of people who have been diagnosed as being on the autistic spectrum. I would, however, suggest that these representations are likely to both reflect and contribute to shape the ‘folk’ models or general schemata of autism shared by readers. In the extracts I have discussed, the characters’ difficulties with communication tend to be presented as relatively harmless, and, as I have shown, sometimes rather humorous, at least from the readers’ perspective. On the other hand, they are also one of the signals of the characters’ potential vulnerability in social relations, which causes all three characters some real hardships as each plot progresses.

I have also mentioned throughout how, by adopting the perspective of the characters with ASDs, the three authors defamiliarise everyday conversational interaction, potentially resulting in some degree of ‘schema refreshment’ (Cook, 1994): readers are put in a position to become more consciously aware of how much is taken for granted in apparently simple, routine exchanges, and of how disconcerting and treacherous these conversations can be for someone who finds other people relatively unreadable. Because, as readers, we see things through the viewpoint of the character with ASDs, we are also privy to the confusion and doubts that underlie their conversational behaviour, which, seen from outside, could lead to a rather more negative evaluation.

Thomas (1983) points out how pragmatic failure can lead to negative value judgements in communication between native and non-native speakers of a particular language. Landa (2009: 125) comments that ‘[p]ragmatic impairment may be the most stigmatizing and handicapping aspect’ of autism and Asperger syndrome. In contrast, in the three novels I have discussed, pragmatic failure arguably makes a contribution to a generally sympathetic portrayal of each protagonist. None of the three characters is idealised as perfect or saintly, and Christopher in particular causes considerable heartache to his parents in the course of the novel. Overall, however, all three protagonists are presented positively, and their conversational difficulties are likely to encourage readers to sympathise with them. More generally, all three novels more or less explicitly contribute to broader debates about the position within society of people with ASDs, and of people with special needs or disabilities more generally (see also Bates, 2010). When talking about *The Curious Incident*, Haddon has said that the novel is not so much about autism as it is about ‘difference, about being an outsider, about seeing the world in a surprising and revealing way’ (Haddon, 2009). All three novels draw attention to the potential for misunderstanding and offence that ‘normal’ people tend to avoid successfully, and to the compromises that communicating ‘normally’ involves between honesty and social harmony. The characters with ASDs are therefore presented as conversational underdogs, while other characters sometimes appear to be vague or ambiguous, if not downright deceptive and hypocritical. In other words, amongst other things, these novels reveal the hidden complexities of communication that people are normally not explicitly conscious of, and raise awareness about the genuine puzzlement that may be experienced by somebody who does not share in the large amounts of knowledge and meanings that tend to remain unspoken.

Finally, my analyses point out that the notion of ‘pragmatic failure’ is not only relevant to the difficulties experienced by non-native speakers communicating in a culture different from their own, but can usefully be extended to the difficulties that are experienced by anyone (whether real or fictional) who does not share in the knowledge and mind-reading abilities that are normally taken for granted in conversation. While I have somewhat stretched Thomas’s (1983) definition, I have found pragmatic failure to be a useful umbrella term for the communicative problems that occur in the three novels I have analysed.

## Notes

1. Fictional characters with recognisably autistic traits pre-date the scientific description of ASD however, as in the case of Bartleby in Melville’s *Bartleby, The Scrivener* and Boo Radley in Harper Lee’s *To Kill a Mocking Bird* (Bates, 2010; Fanlo Piniés, 2005).

2. Although Theory of Mind (ToM) problems are not equally central to different theories of ASD, Cummings (2009: 123) suggests that ‘the claim that “there are ToM *deficits* in autism” […] would be difficult to deny’ regardless of the theoretical account one subscribes to.

3. Christopher’s expression of his own age, and arguably other references to time in both cases, also seem to breach Levinson’s M-Principle: ‘Indicate an abnormal, non-stereotypical situation by using marked expressions that contrast with those you would normally use to describe the corresponding normal, stereotypical situation.’ (Levinson, 2000: 136). There is no evidence to suggest that Lou and Christopher were indicating anything abnormal or non-stereotypical when making unusually detailed references to time or age.
